# Physics-based lung collapse simulation for accurate intraoperative localization during thoracoscopic surgery

**DOI:** 10.1016/j.xjon.2025.11.009

**Published:** 2025-11-19

**Authors:** Yosuke Matsuura, Mitsue Kawahara, Sodai Nagata, Ayumi Suzuki, Junji Ichinose, Masayuki Nakao, Mingyon Mun

**Affiliations:** Department of Thoracic Surgical Oncology, Cancer Institute Hospital, Japanese Foundation for Cancer Research, Tokyo, Japan

**Figure:**
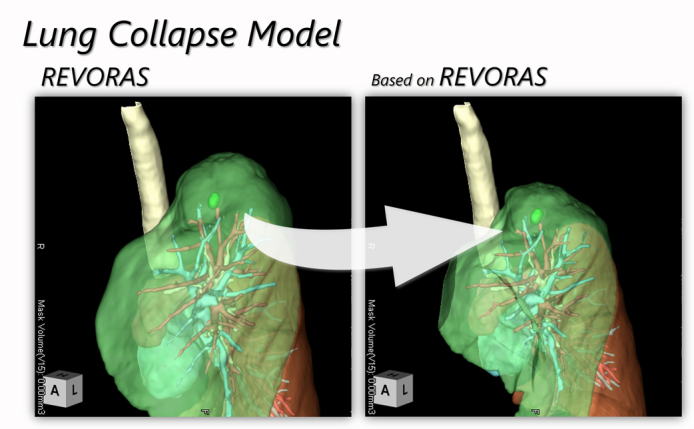
Physics-based lung collapse model aligns with thoracoscopic tumor localization.

**Central Message**

The physics-based lung collapse model accurately predicts tumor location during thoracoscopic wedge resection, enabling precise intraoperative navigation, potentially improving surgical outcomes.

Thoracic surgery relies on preoperative computed tomography (CT) reconstructions. However, intraoperative lung collapse nonlinearly deforms anatomy, reducing navigation accuracy. Dynamic simulation systems such as the Resection Process Map^1^ and shape-matching analyses^2^ have emphasized the need for biomechanical modeling of intraoperative anatomy. We developed and validated a physics-based lung collapse model using the REVORAS platform (Ziosoft) to predict intraoperative tumor localization during wedge resection.

## Methods

**Figure 1:**
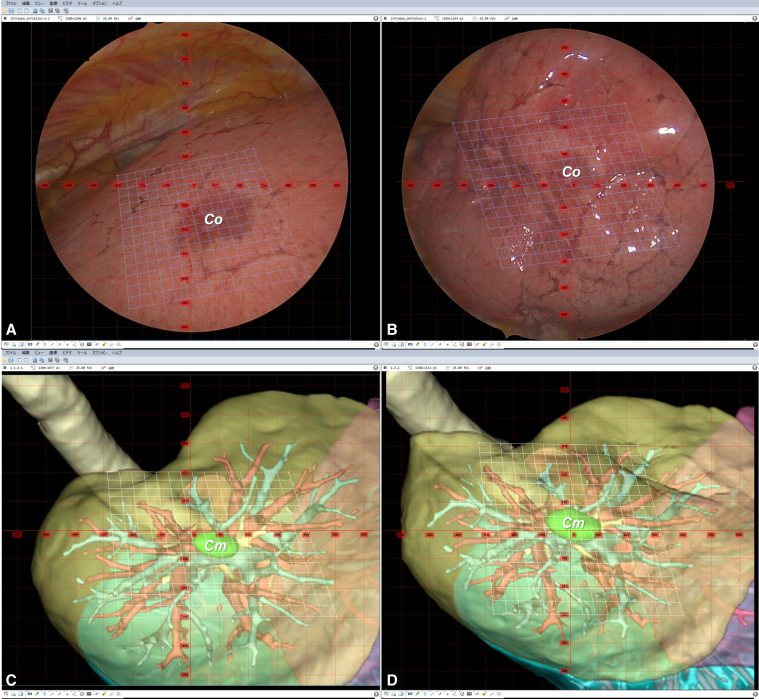
Comparison between thoracoscopic and simulated deflated lung images. A and B, Thoracoscopic views showing the deflation process. C and D, Corresponding physics-based simulated models illustrating lung collapse and target nodule displacement (*green sphere*). In quantitative evaluation, 3 surgeons rated model-view concordance on a 5-point scale. In quantitative evaluation, tumor centers in the intraoperative video (Co) and model predictions (Cm) were compared. The Euclidean distance *d(Co, Cm)* was calculated per case. Motion fidelity was evaluated by mean squared error between Co and Cm.

This retrospective single-center study was institutional review board approved (approval No.: 2023-GB-078; date: October 11, 2023). Thirteen patients who underwent wedge resection provided written consent for publication (see Figure 1).

The model used an algorithm with a virtual deflation process assuming uniform contraction. Preoperative CT data were processed on the REVORAS platform to generate 3-dimensional models of inflated and deflated states. Boundary conditions and contraction coefficients were optimized using intraoperative observations. The system was developed through a joint research collaboration between our institution and Ziosoft Inc. Qualitative evaluation: 3 thoracic surgeons rated model-view concordance on a 5-point scale (1 = very poor, 5 = excellent).

Quantitative evaluation: Tumor centers from intraoperative video (Co) and model predictions (Cm) were compared. The Euclidean distance, representing the shortest distance between points in 3-dimensional space, was calculated for each case:

$$d\left(Co,Cm\right)=\sqrt{{\left(x_{o}-x_{m}\right)}^{2}+{\left(y_{o}-y_{m}\right)}^{2}}$$

Motion fidelity was assessed by mean squared error (MSE) between predicted and observed displacement vectors:

$$MSE=\frac{1}{n}\sum_{i=1}^{n}\left[{\left(x_{o,i}-x_{m,i}\right)}^{2}+{\left(y_{o,i}-y_{m,i}\right)}^{2}\right]$$

## Results

Tumor locations are shown in Table 1. Mean surgeon ratings were 4.0, 4.1, and 3.3 (overall, 3.8), indicating good visual concordance. The mean localization error was 1.74 ± 0.22 mm (range, 1.45-2.26 mm), and 12 of 13 lesions were localized within 2.0 mm. The mean displacement MSE was 3.06 × 10^−5^ mm^2^, demonstrating high motion fidelity.

**Table 1:** Qualitative and quantitative results

Case	Tumor location	Qualitative evaluation∗			Quantitative evaluation				
		Surgeon 1 (M.K.)	Surgeon 2 (S.N.)	Surgeon 3 (Y.M.)	$x_{o}$	$y_{o}$	$x_{m}$	$y_{m}$	$d\left(C_{0},C_{m}\right)\;\left[mm\right]$
1	LUL	5	4	5	24.98	18.49	24.51	17.07	1.49
2	LLL	5	4	4	48.03	17.27	49.69	17.42	1.66
3	RLL	5	4	4	39.28	17.34	40.61	18.21	1.59
4	RUL	4	3	4	33.95	22.17	32.32	21.68	1.70
5	RUL	4	5	4	16.24	30.99	17.53	32.02	1.65
6	RLL	4	5	4	16.24	27.28	15.39	25.71	1.78
7	LUL	5	5	4	12.32	21.65	11.00	21.07	1.45
8	RML	3	4	3	44.65	34.47	42.83	35.08	1.92
9	RLL	3	4	3	38.32	21.69	37.63	23.50	2.26
10	RLL	3	3	4	10.82	24.65	11.41	23.01	1.94
11	RLL	4	4	4	10.82	24.65	11.41	23.01	1.74
12	RLL	4	4	4	48.80	28.24	47.26	27.16	1.88
13	RLL	5	4	4	43.30	41.41	43.90	42.78	1.50
Average		4.0	4.1	3.3	31.61	24.71	31.22	24.68	1.74
			Average of 3 surgeons: 3.8		Displacement MSE: 3.06 × 10^−5^ [mm^2^]				

*LUL*, Left upper lobe; *LLL*, left lower lobe; *RLL*, right lower lobe; *RUL*, right upper lobe; *RML*, right middle lobe; *MSE*, mean squared error.

∗ Evaluation scores were 1 = very poor, 2 = poor, 3 = fair, 4 = good, and 5 = excellent.

## Discussion

The physics-based simulator reproduced intraoperative tumor localization with submillimeter accuracy. Unlike CT-based navigation, this model accounts for nonlinear deformation during deflation, improving the match between preoperative planning and intraoperative findings. Accuracy was comparable to that of previous deformation analyses,^3^ supporting the feasibility of simulation-assisted navigation for precise tumor localization and parenchymal preservation.

The model was simplified to be uniform and did not account for time-dependent changes. This provided robust predictions within 2-mm accuracy. The qualitative assessment involved 3 surgeons, including the first author, under blinded conditions. Single proprietary platform (REVORAS) use may limit generalizability, but the approach is transferable to other systems.

## Conclusions

This model achieved <2 mm error, demonstrating feasibility for intraoperative navigation. Prospective validation and extension to segmentectomy are required.

## Conflict of Interest Statement

The authors reported no conflicts of interest.

The *Journal* policy requires editors and reviewers to disclose conflicts of interest and to decline handling or reviewing manuscripts for which they may have a conflict of interest. The editors and reviewers of this article have no conflicts of interest.
